# Deposition of Zinc Oxide on Different Polymer Textiles and Their Antibacterial Properties

**DOI:** 10.3390/ma11050707

**Published:** 2018-04-30

**Authors:** Marta Fiedot-Toboła, Magdalena Ciesielska, Irena Maliszewska, Olga Rac-Rumijowska, Patrycja Suchorska-Woźniak, Helena Teterycz, Marek Bryjak

**Affiliations:** 1 Faculty of Microsystem Electronics and Photonics, Wrocław University of Science and Technology, Janiszewskiego 11/17, 50-372 Wrocław, Poland; olga.rac-rumijowska@pwr.edu.pl (O.R.-R.); patrycja.wozniak@pwr.edu.pl (P.S.-W.); helena.teterycz@pwr.edu.pl (H.T.); 2 Faculty of Chemistry, Wrocław University of Science and Technology, Norwida 4/6, 50-373 Wrocław, Poland; magdalena.ciesielskaa@gmail.com (M.C.); irena.helena.maliszewska@pwr.edu.pl (I.M.); marek.bryjak@pwr.edu.pl (M.B.)

**Keywords:** zinc oxid, polymer textiles, nanoparticles, microrods, wettability, roughness, antimicrobial properties

## Abstract

A surface modification of polyamide 6 (PA), polyethylene terephthalate (PET) and polypropylene (PP) textiles was performed using zinc oxide to obtain antibacterial layer. ZnO microrods were synthesized on ZnO nanoparticles (NPs) as a nucleus centers by chemical bath deposition (CBD) process. Scanning Electron Microscopy (SEM) and X-ray diffraction (XRD) indicated that wurzite ZnO microrods were obtained on every sample. Differential Scanning Calorimetry (DSC), Fourier Transform Infrared Spectroscopy (FTIR), Atomic Force Microscopy (AFM) and Liquid Absorption Capacity (LAC) analysis indicate that the amount and structure of antibacterial layer is dependent on roughness and wettability of textile surface. The rougher and more hydrophilic is the material, the more ZnO were deposited. All studied textiles show significant bactericidal activity against *Escherichia coli* (*E. coli*) and *Staphylococcus aureus* (*S. aureus*). A possible mechanism and difference in sensitivity between Gram-negative and Gram-positive bacteria to ZnO is discussed. Considering that antibacterial activity of ZnO is caused by Reactive Oxygen Species (ROS) generation, an influence of surface to volume ratio and crystalline parameters is also discussed.

## 1. Introduction

The textile industry, similar to other sectors, systematically changes as a result of new product development which meets user expectations, as well as the enrichment of textile applications in various fields. Nowadays, there are many functional textiles but the most popular of them seem to be materials with antibacterial properties [1,2,3]. High demand for this type of fabric is related to the problem of increasing resistance of microorganisms to drugs [4,5]. Antimicrobial materials can help in preventing reproduction and spread of pathogens. Therefore, they could be used for obtaining disposable textiles such as protective aprons, caps, surgical curtains, and dressings, as well as reusable materials such as bed sheets, towels or work clothes. They could also be used in everyday applications, such as sport clothes to stay fresh longer.

On the textile market there are many antibacterial agents. They differ not only in effectiveness and durability but also in impact on users and the environment. The most common substances include ammonium salts [6,7], chitosan [8,9], triclosan [10,11] and metal or metal oxide nanoparticles [12,13,14].

In this paper, the modification of three different types of fabrics (PA, PET, and PP) with zinc oxide microrods is presented. These fabrics are commonly used in the textile industry and are made of polymers of varying surface wettability. ZnO has been selected as the antibacterial agent because it is non-toxic, very effective and does not change the color of the output fabrics [15,16]. In previous studies authors have shown that the morphology and crystalline structure of ZnO is crucial for the final antimicrobial properties [17,18]. In this work, zinc oxide microrods were not grown directly on the fabric surface, but rather colloidal ZnO nanoparticles were used as crystallization nuclei. The authors suppose that this allows for a significant increase in biological activity of the modified fabrics. Obtained antibacterial materials could be used in everyday life applications such as sport clothes, medical textiles or bedding.

## 2. Materials and Methods

### 2.1. Applied Textile Materials

In these studies, three different textiles were used and modified: PA, PET and PP. The first two were made by WISTIL S. A. company from Kalisz, Poland (product No 5707, 5235). The PP textile was manufactured by Łódź University of Technology (Łódź, Poland). The aforementioned materials are composed of different polymer groups: polyesters, polyamides and polyolefins. These textiles have similar structure and high resistance to deformations. They were made in 1/1 plain weave with thickness ca. 0.20 mm. The average size of macro-pores in the textiles ranged from 30 to 70 µm. Textiles were initially washed and thermally stabilized after production (Table 1).

To analyze properties of inspected textiles, several measurements were performed. The surface was studied by a Scanning Electron Microscope Evo LS 15 (Zeiss, Oberkochen, Germany) and an Atomic Force Microscope. AFM measurements were carried out using a commercial NanoMan V microscope with a NanoScope V Controller (Veeco Instruments/Bruker, New York, NY, USA). Samples were scanned in tapping mode with spring constant 40 N/m and resonant frequency 300 kHz using RTESP probes (Veeco Instruments/Bruker, New York, NY, USA). Acquired data were processed using non-commercial software TopoGraf (Faculty of Microsystem Electronics and Photonics, Wrocław University of Science and Technology, Wrocław, Poland). Subsequently, the root mean square roughness (Rq) was calculated.

Differential Scanning Calorimetry studies were performed using DSC 6 equipment (Perkin Elmer, Waltham, MA, USA). These tests were performed in nitrogen atmosphere, passing through the system at a rate of 20 mL/min. The analysis of the obtained results was performed using Pyris software (v.3.81, Perkin Elmer, Waltham, MA, USA). The crystallinity degree was also calculated.

The chemical structure of samples was examined using Fourier Transform Infrared Spectroscopy in the Nicolet 380 system (Thermo Fisher Scientific, Waltham, MA, USA). The spectrum was recorded in the range of 60 to 4000 cm^−1^ with a resolution of 4 cm^−1^.

The wettability of textiles was measured as an ability to absorb liquid, in accordance with ISO 9073-6:2003 standard “Textiles. Test methods for nonwovens. Part 6: Absorption” [19]. Materials were cut into 10 × 10 cm^2^ samples, and then weighed before and after immersing in water for 60 s.

### 2.2. Textile Modification Process

To induce antibacterial properties in textiles, zinc oxide microrods were deposited on their surface by hydrothermal method. Before the process, materials were dipped in colloidal zinc oxide nanoparticles for 30 s and then dried at 90 °C for 15 min. The colloid contained a spherical ZnO nanoparticles with average radius of ca. 6 nm. Their characterization and synthesis method were presented in previous work [20]. The nanoparticles adsorbed on the textile surface during hydrothermal ZnO microrods deposition were nucleation centers. The hydrothermal deposition was carried in a 100 mM water mixture of hexamethylenetetramine (HMT) and zinc nitrate (Zn(NO_3_)_2_·6H_2_O). Obtained chemical bath was stirred for 24 h and filtered. Then, textile samples were added and the whole system was heated to 90 °C for 8 h. After the deposition process, modified samples were washed in ultrasound washer EW-08849-02 (Cole-Parmer, Vernon Hills, IL, USA) and dried in a room temperature.

The next step was to characterize obtained antibacterial layer. Mass changes of samples were determined, their microstructure and crystal structure were examined using accordingly SEM and X-ray Philips Materials Research Diffractometer (Philips, Eindhoven, Netherlands), utilizing CuKα radiation. Obtained results include: interplanar distance, lattice constants and the volume of the unit cell, determined using Bragg’s law and the Williamson–Hall (W-H) method.

The antibacterial properties of modified textiles were determined by the serial dilution method against two different pathogens: Gram-negative *E. coli* PCM 2057 and Gram-positive *S. aureus* PCM 458. An amount of 1 mL of an overnight culture of the tested bacteria (grown aerobically at 37 °C, with shaking, in Mueller broth) was centrifuged at 5000 g for 5 min, and then the supernatant was discarded. The pellet was re-suspended in 1 mL of saline to give an inoculum of approximately 1.2 × 10^6^ colony-forming units (CFU/mL) for *S. aureus* and 1.3 × 10^7^ CFU/mL for *E. coli*. Samples of the ZnO modified textiles (1 cm × 1 cm) were placed in tubes containing 3 mL of distilled water. Then, 300 (or 30) μL of pre-prepared bacterial suspension was added to the tubes. Tubes were incubated for 2, 5 and 24 h at 37 °C. Then, serial dilutions were prepared and 100 µL aliquots of each dilution were seeded in duplicate onto Mueller Agar (Difco) and incubated for 24–48 h at 37 °C. After incubation, the number of colony forming units per milliliter (CFU/mL) was established. Bacterial suspensions treated with unmodified PA, PET and PP as well as suspensions without textile samples were positive and negative controls, respectively.

The antibacterial activity of modified textiles was also studied on the solid medium in accordance with the ISO 20645:2004 standard “Textile fabrics -Determination of antibacterial activity. Agar diffusion plate test” [21].

## 3. Results and Discussion

### 3.1. Textile Characterization

**Figure 1:**
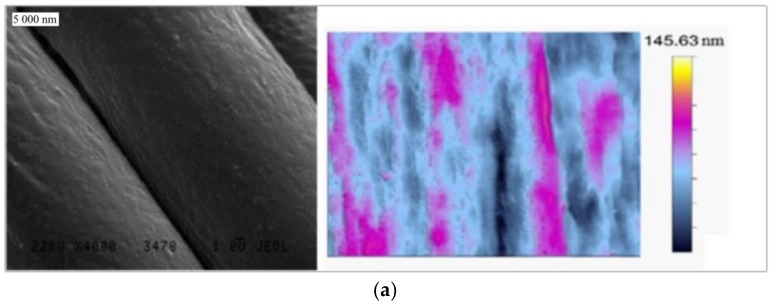

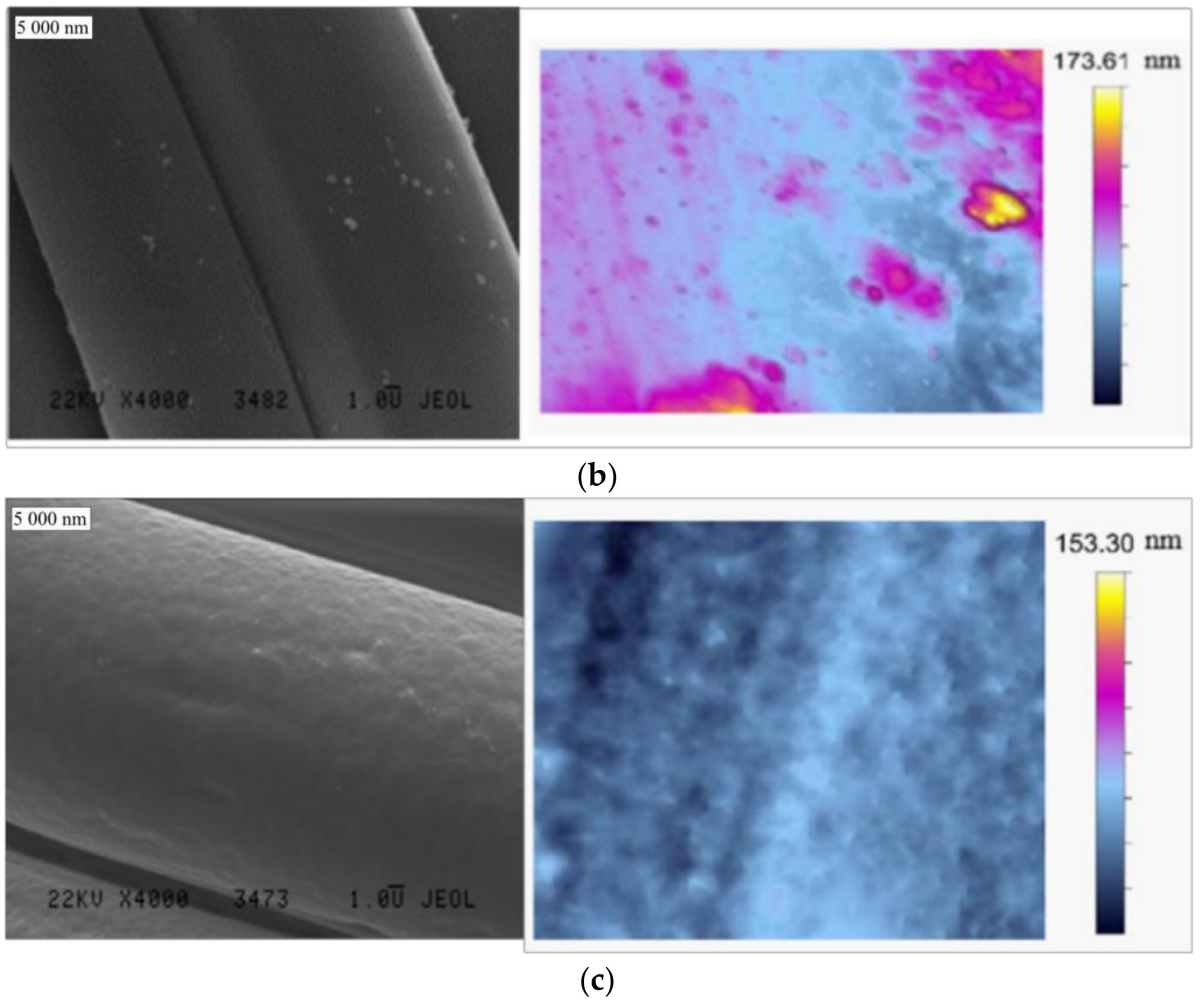
SEM and AFM images of: (**a**) PA; (**b**) PET; and (**c**) PP.

Based on SEM and AFM observations, it was indicated that the surface of PET is smooth but rich in particles which could be residues from the production process (Figure 1b). PP and PA surfaces were rough and free of pollutions (Figure 1a,c). Those results were confirmed by a root mean squared value which was 17.98 nm for PA, 6.57 nm for PET and 10.50 nm for PP.

**Figure 2:**
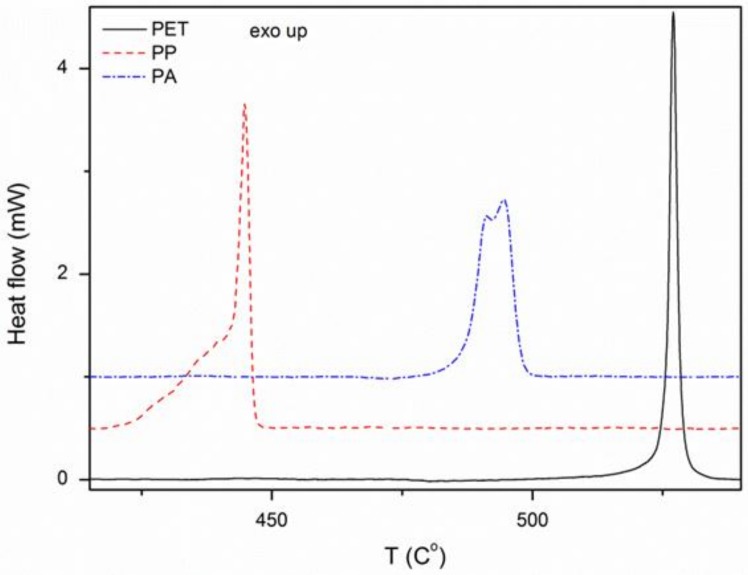
DSC analysis of the used textiles.

The composition and degree of crystallinity of the materials were determined based on DSC analysis. These studies have shown that fabrics consist exclusively of polymers, as declared by manufacturers. This is indicated by the presence of characteristic exothermic peaks (Figure 2). The degree of crystallinity was calculated using Formula (1) and summarized in Table 2. It was observed that *X_c_* of all textiles is similar.

(1) $$X_{C}=\frac{\Delta H_{m}}{\Delta H_{m100}}$$

where Δ*H_m_* is the melting enthalpy of sample and Δ*H_m_*_100_ is the melting enthalpy of 100% crystalline polymer.

**Figure 3:**
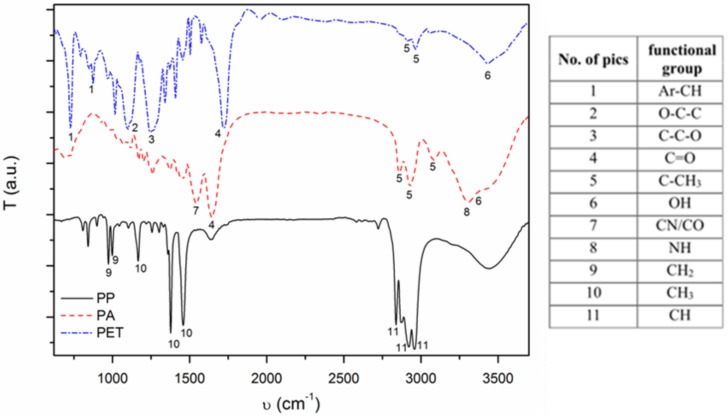
FTIR results for analyzed textiles.

FTIR analysis confirmed DSC results, showing peaks characteristic for functional groups of main textile polymers, without any additives (Figure 3) [22,23,24].

The liquid absorption capacity (LAC) was determined according to polish standard ISO 9073-6:2003 Formula (2) [19]. It was observed that the liquid absorption capacity is significantly larger for PA sample than for other materials. Considering standard deviation of the results, it could be seen that there is small difference between PET and PP. However, PP is characterized by slightly smaller LAC value (Table 3), which is a result of its chemical composition. It is built from nonpolar groups containing carbon–hydrogen bonds, whereas PET has oxygen groups and PA has additional amide groups, which are characterized by strong polar properties.

(2) $$LAC=\frac{m_{n}-m_{k}}{m_{k}}$$

where *m_n_* is the mass of wet sample (g) *m_k_* is the mass of dry sample.

### 3.2. Deposition and Characterization of Antibacterial Material

**Figure 4:**
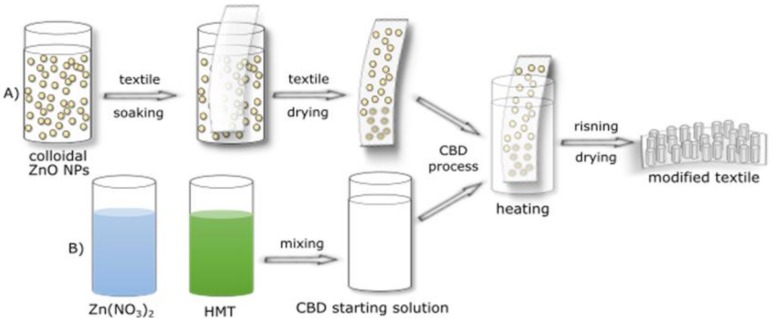
A scheme of modification process. (**A**) ZnO NPs deposition; (**B**) CBD solution preparation.

To form nucleation centers on the textile surface, all fabrics were immersed in a previously prepared ZnO NPs colloid and then dried (Figure 4a). Further, a hydrothermal deposition of ZnO microrods was performed on such materials, using aqueous solutions of zinc nitrate and urotropine. The process was carried out at 90 °C for 8 h (Figure 4b). After the process, the modified fabrics were rinsed in an ultrasonic scrubber to remove the excess of antibacterial agent and then dried at room temperature.

The mechanism of hydrothermal deposition of ZnO using HMT and Zn(NO_3_)_2_ is very complex and multistage. Depending on the temperate and time, different reactions, which determinate obtaining products, take place. Used process condition (90 °C, 8 h) were selected based on previous studies on the influence of temperature and time on zinc oxide hydrothermal deposition [17,18]. For this reason, these topics are not discussed in this article.

The amount of zinc oxide deposited on the substrate was determined by measuring the weight gain. These measurements have shown that the more hydrophilic is the fabric, the more zinc oxide is deposited on the surface. Weight gain was accordingly 6.3 ± 0.2 mg for PA, 5.1 ± 0.1 mg for PET and 3.9 ± 0.1 mg for PP. 

**Figure 5:**
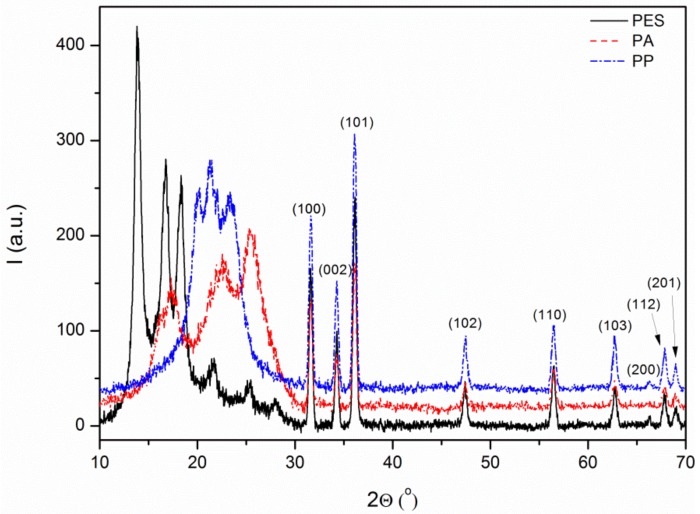
XRD diffractograms of modified textiles.

The crystal structure analysis of the antibacterial layer formed on the fiber surface was performed using XRD. Characteristic packs were observed on the obtained diffractograms at about 2*θ* = 31.6°, 34.2°, 36.1°, 47.4°, 56.5°, 62.7°, 65.7°, 67.5°, and 69.0°, which corresponds to the crystal planes [100], [002], [101], [102], [110], [103], [200], [112], and [201] of hexagonal wurzite structure that were observed (Zincite, JCPDS 5-0664). That confirmed the presence of ZnO on all textile surfaces (Figure 5).

**Figure 6:**
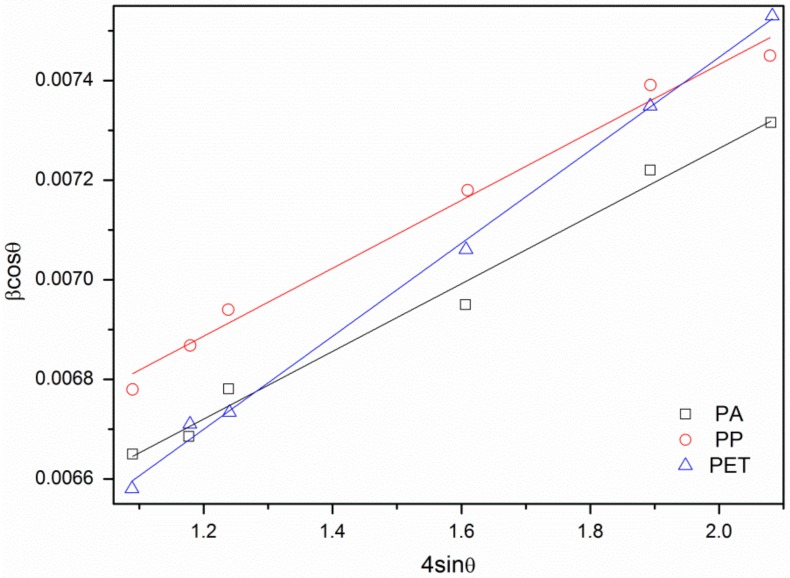
The W-H curves for samples.

To perform a thorough characterization of obtained ZnO crystals, interplanar distances, lattice constants, and the unit cell volume were calculated [25]. It was observed that the values of lattice constants and the unit cells volume are similar for all samples and are bigger than the standard values for zinc oxide cell (Table 4). The microstrains and average crystallite size in ZnO were also determined, using the Williamson–Hall (W-H) method [26]. To calculate the strain curve and crystallite size, *β*cos*ϕ* = f(4sin*ϕ*) was plotted for preferred diffraction peaks (Figure 6).

It was observed that the crystallite size is increasing for samples in order PET, PA and PP. These results, concerning the strain values in ZnO structure show the same tendency. A conclusion could be made that the bigger is the crystallite size, the bigger is the strain observed. Comparison between these results and standard data (Zincite, JCPDS 5-0664) showed that every obtained crystallite was characterized by greater unit cell parameters values than data determined by JCPDS (Table 4). This could be a consequence of defects embedded in the structure, amount of which is related to strain values and crystallite size. The most irregular crystals were obtained for ZnO deposited on PP surface. The results for PA and PET were very similar. Therefore, authors suppose that, in the substrate, hydrophobicity plays the most important role the crystallization process.

**Figure 7:**
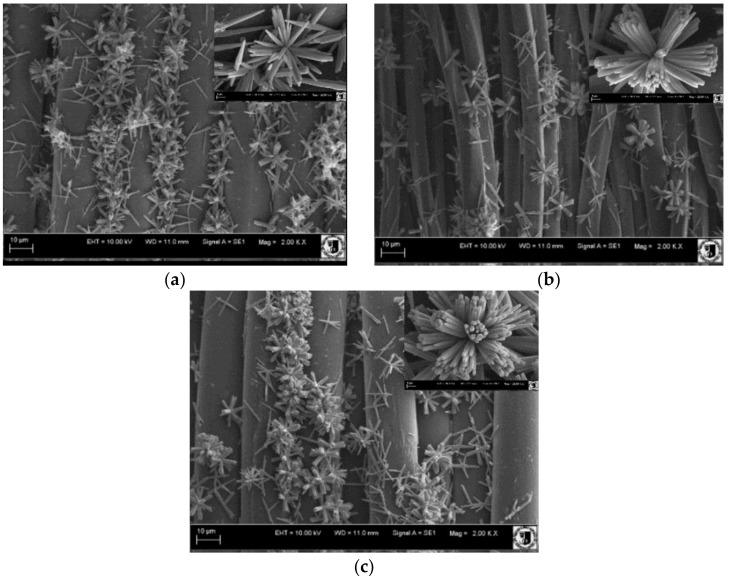
SEM images of ZnO deposited on: (**a**) PA; (**b**) PET; and (**c**) PP.

The microstructure of ZnO deposited on the surface of polymer materials was determined by SEM observation. The elongated zinc oxide structures formed by flower-like agglomerates were created (Figure 7). The agglomerates on the surface of the PA are made of sharp-ended single rods, while those on PP and PET are made of microrods. The average length of the ZnO microrods grown on the surface of PA, PET, and PP materials was accordingly 3.9 ± 0.4, 5.6 ± 0.2, and 4.1 ± 0.6 μm, respectively. Their diameters are 252 ± 5, 398 ± 8, and 313 ± 2 nm, respectively. Based on these values, calculated average shape ratio (A_R_) [17] was 15.6, 14.3, and 13.2, respectively.

**Figure 8:**
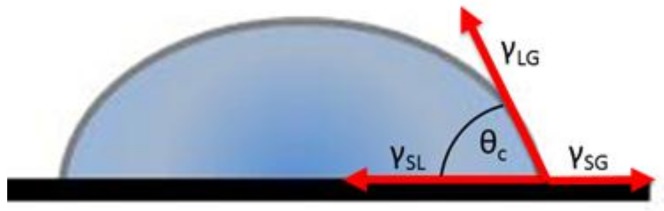
A scheme of a of contact angle determination.

The shape, location and crystalline structure of ZnO rods are significantly different from the results of the authors’ previous work [17]. In this study, a relatively even distribution of zinc oxide rods on the surface of the fabric, without agglomerates, was observed. These structures have a similar length but are much thicker (1.6 μm in diameter). Authors believe that this is because ZnO nanoparticles are used in the present study as heterogeneous nucleation centers. The heterogeneous nucleation occurs much more easily on the surface of the solid-state nanoparticles in contact with the solution than homogeneous nucleation directly from solution. If the crystallization nucleus is presented as a sphere, the equation describing the equilibrium of surface energy at the triple point follows Equation (3) (Figure 8). Because crystallization nucleus is made of ZnO NPs, on which ZnO crystals are growing, the surface energy between ZnO nucleus and liquid (*γ_NL_*) is equal to surface energy between liquid and ZnO crystal (*γ_LC_*) (4–6).

Therefore, the reduction of the energy required for the crystallization of the material in the heterogeneous process compared to the homogeneous depends on the contact angle of the material and is expressed by the Volmer coefficient *f*(*θ*) in Equation (7). Thus, the smaller is the contact angle of the material at which crystallization occurs, the lower is the energy required for this process [27].

(3) $$\gamma_{NL}=\gamma_{NC}+\gamma_{LC}\cos\theta$$

(4) $$\gamma_{NL}=\gamma_{LC}$$

(5) $$\gamma_{NL}-\gamma_{LC}\cos\theta =\gamma_{NC}$$

(6) $$\gamma_{NL}(1-\cos\theta )=\gamma_{NC}$$

(7) $$f\left(\theta \right)=\frac{\left(2+\cos\theta \right)\cdot {\left(1-\cos\theta \right)}^{2}}{4}$$

where *γ_NL_* is the surface energy between nucleus and liquid (J/m^2^); *γ_NC_* is the surface energy between nucleus and crystal [J/m^2^]; *γ_LC_* is the surface energy between liquid and crystal (J/m^2^); and *θ* is the contact angle (^o^).

In the present studies, crystallization was carried out on nanoparticles of ZnO applied on the fabric surface. Consequently, the ZnO crystallization energy on the surface of all three fabrics should be comparable. However, it should be significantly smaller than in the case of using textiles not covered by ZnO nanoparticles (without crystallization nucleus). Therefore, the first stage of the process (the deposition of ZnO nanoparticles on the textile surface) has a decisive influence on the formation of zinc oxide rods.

The LAC, R_q_, SEM and AFM analyses provide the most important surface parameters for the evaluation of nanoparticle influence on the fiber surface. LAC value demonstrates the ability to absorb solvent and is therefore connected with both surface chemistry and roughness. R_q_ is a numerical indicator of surface roughness. Comparison of those two parameters can provide all necessary information about textile surface. Based on obtained results, the LAC and R_q_ values for PA are the highest. PET and PP have similar LAC value but much smaller than PA. The R_q_ value was bigger for PP than for PET. Those results were confirmed by SEM and AFM images. The PET surface is flat, but the PA and PP surface have “orange peel” structure. Those observations, in combination with DSC and FTIR spectra, lead to conclusion that PA surface is the roughest and most hydrophilic. Surface of the PET fibers are flat and hydrophilic, while the PP surface is rough and hydrophobic.

**Figure 9:**
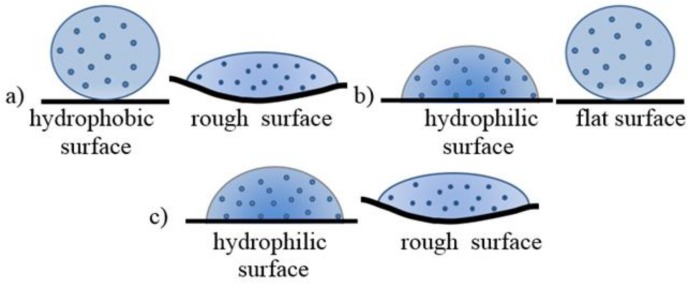
A shape of colloid drop on different textile surfaces: (**a**) PP; (**b**) PET; and (**c**) PA.

During immersion of substrates in zinc oxide colloid, nanoparticles can bind with the textile by chemisorptions, physisorption and/or hydrogen bond on the surface. The effectiveness of this bond depends on wettability and surface roughness. In the case of rough hydrophobic surfaces (Figure 9a) or on flat hydrophobic surfaces (Figure 9b), the crystallization centers can be created only in some places with limited area. On the other hand, on the hydrophilic and rough fibers, nanoparticles could be easily adsorbed (Figure 9c).

**Figure 10:**
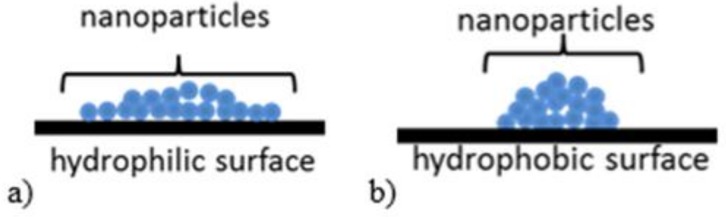
A scheme of the ZnO NPs on: (**a**) hydrophilic; and (**b**) hydrophobic surface after drying.

The amount of ZnO deposited on the textile surface is decreasing in following order: PA, PET, and PP. The crystallite size and strain values of ZnO are increasing in the order: PET, PA and PP. In addition, some differences were observed on SEM images (Figure 7). On PA, textile needle like structures were observed with the highest A_R_ value (3.9 µm length, 252 nm diameter). On PET, the longest rods were deposited with higher A_R_ (5.6 µm length, 398 nm diameter) than on PP sample (4.1 µm length, 313 nm diameter). In addition, the zinc oxide microcrystals formed flower-like agglomerates on the fabric surface. Agglomerates formed on the surface of the PA are made of sharp-ended rods. In contrast, the agglomerates formed on the PP and PET surfaces are constructed of microrods because the fabrics after immersion in the colloidal solution were dried. Nanoparticles tend to agglomerate during drying process as the consequence of increase in concentration. This effect is most easily achieved on smooth and/or hydrophobic surfaces (Figure 10).

Aforementioned nanoparticle agglomerates are base for ZnO microrods growth. As a result, the biggest amount of ZnO nanoparticles is present on PA surface, then on PET and the least on PP. It has to be noticed that the difference between PET and PP is not significant, which is due to the balance between roughness and wettability of their surfaces. After shape, crystallite size and structure strains analyses a conclusion could be made that on PA nucleation is preferred to the growth process, in contrast to PET. This argument is affirmed by needle like crystals observed in sample of PA (Figure 7a) which are the consequence of Zn^2+^ deficiency. On PP, nanoparticles were loosely attached to the surface, due to its hydrophobic character and the fact that the size of colloid drops is determined only by surface roughness. This is proven by the biggest strain values in ZnO microrods on PP textile.

### 3.3. Antibacterial Characterization

**Figure 11:**
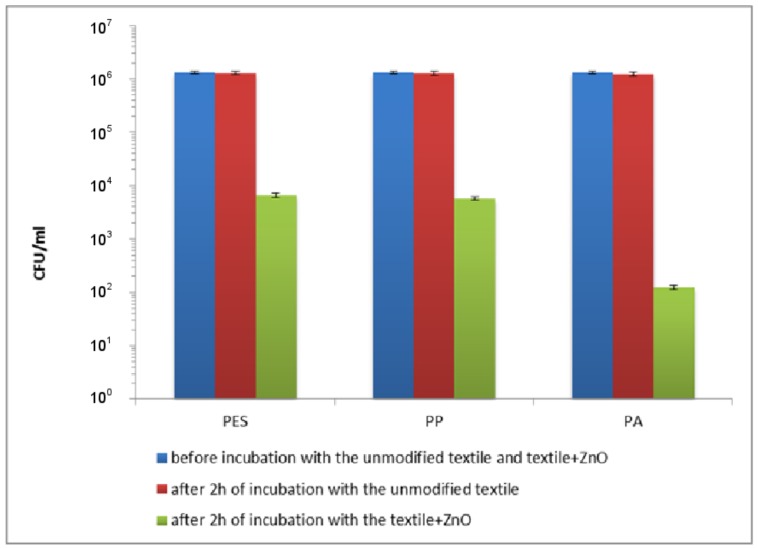
The effect of the modified polymer textiles on the viability of *E. coli* determined by the serial dilution method.

**Figure 12:**
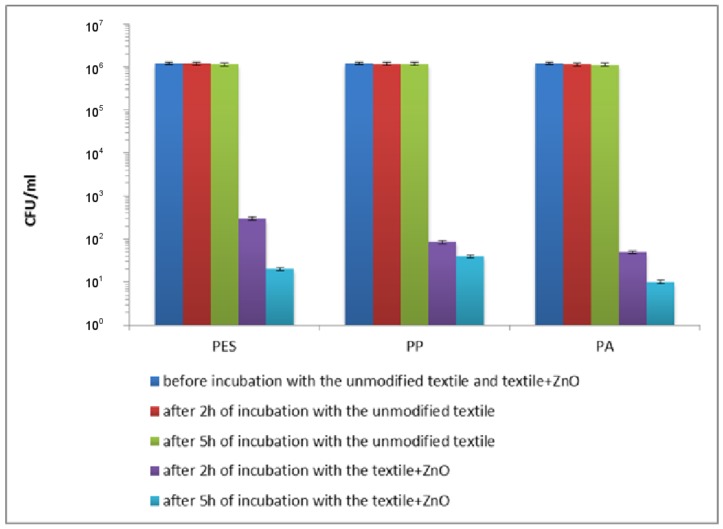
The effect of the modified polymer textiles on the viability of *S. aureus* determined by the serial dilution method.

The bactericidal activity of the textile and control samples was assessed using *Escherichia coli* and *Staphylococcus aureus*. The decrease in the number of *E. coli* after 2 h of incubation in saline (data not shown) and in the presence of the unmodified textiles (Figure 11) was similar and ranged 2 × 10^4^–5 × 10^4^ CFU/mL for PES, PP and PA. The decrease in the number of *S. aureus* after 5 h of incubation in saline (data not shown) and in saline with the unmodified textiles (Figure 12) was also similar and ranged 3 × 10^4^–7 × 10^4^ CFU/mL for PP, PES and PA. These experiments confirmed that unmodified textiles did not show the antibacterial activity against *E. coli* and *S. aureus*. The insignificant decrease in the number of bacteria was the result of un-growing conditions of the experiments. Appreciable destruction of planktonic rods and coccus was achieved using the textiles modified by ZnO. A viability reduction of the studied strains depends on the time of exposure. 

As can be seen in Figure 11, after 2 h of incubation with the modified polymer textiles, the viable count of *E. coli* showed a reduction of 2.36 log_10_ (that is 99.56%), 2.41 log_10_ (that is 99.61%) and 4.08 log_10_ (that is 99.992%) for PET + ZnO, PP + ZnO and PA + ZnO, respectively. A longer exposure time (5 h) resulted in a lethal effect (the count was below the detection limit).

A very high reduction in the number of live cells of *S. aureus* was observed after 2 h of exposure to the studied textiles (Figure 12). It was found that reduction in viability of cells was 3.60 log_10_ (that is 99.98%), 4.15 log_10_ (that is 99.99%) and 4.38 log_10_ (that is 99.996%) for PET + ZnO, PP + ZnO and PA + ZnO, respectively. When *S. aureus* was treated for 5 h with the studied samples, the reduction in the number of bacterial cells was 4.78 log_10_ (that is 99.998%), 4.48 log_10_ (that is 99.997%) and 5.08 log_10_ (that is 99.999%) for ZnO coated PET, PP and PA (Figure 12), respectively. Extending the exposure time to 24 h caused total mortality of bacteria (the count was below the detection limit).

**Figure 13:**
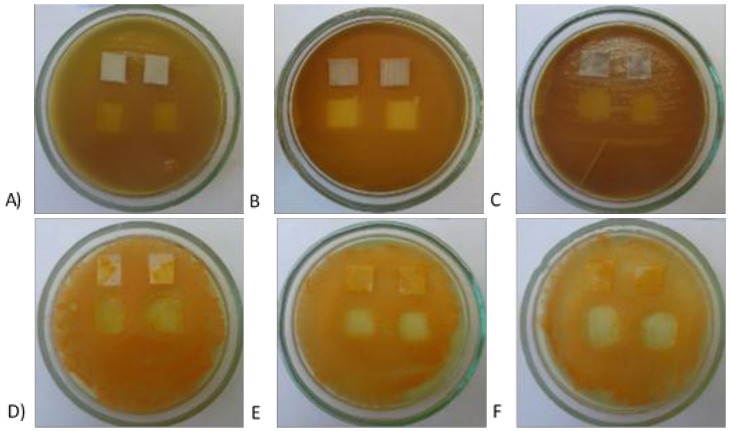
The effect of the modified polymer textiles on the viability of *E. coli* (PET-ZnO (**A**); PA-ZnO (**B**); and PP-ZnO (**C**)) and *S. aureus* (PET-ZnO (**D**); PA-ZnO (**E**); and PP-ZnO (**F**)) determined by the plate method.

Antimicrobial activity of the modified PET, PP and PA has also been studied on the solid medium in accordance to ISO 20645:2004 standard [21]. The following pictures (Figure 13) show clear zones of growth inhibition of *E. coli* and *S. aureus*. The obtained results confirmed that all textiles are characterized by a significant bactericidal activity. It seems that the clearest zones of growth inhibition were observed for PA-ZnO, confirming the excellent bactericidal activity of this textile.

Generally, the studied modified polymer textiles, regardless of the type, showed a significant bactericidal activity. It was observed that Gram-negative *E. coli* was more sensitive to ZnO and 5 h incubation of cells with the textiles resulted in lethal effect. In the case of *S. aureus* (Gram-positive bacterium), longer contact time with ZnO is required to obtain lethal effect. Our results are consistent with previous studies on antibacterial activity of ZnO against various pathogenic and non-pathogenic microbes showing different sensitivity of bacteria to ZnO particles [28].

The mechanism of antibacterial activity in ZnO particles is complex and not fully understood. It is generally believed that toxicity of zinc oxide is related to: (*i*) the uptake of metal ions (translocation and internalization of particles) into cells; (*ii*) the production of ROS from metal oxides and ions with subsequent oxidative damage of cellular structures; and (*iii*) changes in bacterial membrane permeability (gradual release of lipopolysaccharides, membrane proteins, and intracellular factors) and dissipation of the proton driving force as a result of accumulation and dissolution of membrane-containing nanoparticles.

Some authors suggested that the mechanism regarding ZnO transport to cells explains the differences in the sensitivity of Gram-positive and Gram-negative bacteria. It is believed that the attachment of ZnO particles to the cell surface, their transport inside the cell is different and is attributed to different bacterial membrane structure. Therefore, the thickness, structure and composition of the cell wall play an important role in the interaction of ZnO with bacteria [29]. The cell wall of Gram-positive bacteria is a thick layer of peptidoglycan and contains teichoic and lipoteichoic acids. These acids can chelate Zn^+2^ ion from ZnO and transport inside the cell [30]. Gram-negative bacteria have an outer membrane, placed above the thin layer of peptidoglycan. Additionally, between the outer membrane and the plasma membrane, in the periplasmic space, there is a concentrated gel-like matrix (periplasm). Porins, ion channels present in outer membrane of Gram-negative bacteria, facilitate passive diffusion of ZnO inside the cell. In our research, the average length of the ZnO microrods grown on the surface of textiles ranged from 3.9 ± 0.4 to 5.6 ± 0.2 μm. Their diameters were over 252 ± 5 nm. It is therefore difficult to consider that the transport of such large particles to bacterial cells is possible. It seems reasonable to exclude this mechanism as a bactericidal activity of ZnO particles. 

It is possible that destruction of the cell membrane structure and its leakage can be one of the main mechanisms of bactericidal activity of the studied textiles. The interaction of ZnO with membrane can lead to loss of phospholipid bilayer integrity and leakage of intracellular components from the cell, ultimately leading to cell death. For example, Brayner et al. [31] described the loss of cell membrane integrity as the main cause of the bactericidal effect of ZnO on the *E. coli* cell. 

A number of previous papers indicated that ZnO particles generated ROS, such as superoxide anion (O^2^), hydrogen peroxide (H_2_O_2_) and hydroxide (OH^-^) as a major bactericidal factors. The toxicity of these species includes the destruction of molecules, such as lipids, DNA and proteins. It is true that the production of ROS as the main mechanism for the destruction of microorganisms was recognized under the influence of UV light, but in our previous paper it was shown that this mechanism can also takes place in the dark [18].

Considering the fact that the destructive effect of ZnO on the bacteria can be carried out by formation of free radicals, the differences in polarization of bacterial cells appear to be significant. Gram-negative bacteria are more electro-negative than Gram-positive, therefore, due to their electrostatic interactions, they receive less negatively charged free radicals (e.g., hydroxyl radicals) [32,33]. It has also been found that Gram-negative bacteria have partial regenerative capacity [34]. To combat ROS*, S. aureus* uses several defensive molecules, including catalase (KatA), superoxide dismutases (SodA/M), and the golden pigment staphyloxanthin [35,36].

From our results demonstrated above, it appears that the highest bactericidal activity showed PA + ZnO. This unusually high bactericidal activity may be due to the fact that on the surface of PA was deposited the highest amount of ZnO (weight gain was accordingly 6.3 ± 0.2 mg). A comparison of A_R_ and crystalline parameters between PET, PP and PA leads to the conclusion that PA is characterized by the highest values of these parameters. It is well known that a larger surface ratio results in better photocatalytic properties and oxygen adsorption in the semiconductor. This phenomenon is also related to antibacterial activity, as demonstrated in our previous work [17]. It was also found that due to disturbances in the crystal structure, more oxygen is observed in ZnO, which is responsible for the formation of ROS even in the dark [18]. 

It seems that the key to achieving remarkable bactericidal properties is the synergy between A_R_ and crystalline structure. We believed that, in the first stage of the reaction, the oxygen vacancies in the ZnO structure are more important, thanks to which immediate interactions with bacterial cells are possible. Then, the generated ROS starts to play an important role.

## 4. Conclusions

In presented article, three kinds of textiles (PA, PET, PP) were covered with ZnO layer to obtain antibacterial properties.

To assess the influence of surface roughness and chemistry of used materials on zinc oxide synthesis, SEM, AFM, DSC, FTIR and LAC measurements were performed. SEM and AFM results showed a smooth and rich in pollutants PET surface and rough and free of pollutants PP and PA surfaces. DSC and FTIR demonstrated that used textiles are made of polymers declared by manufacturers. LAC, which is connected both with surface roughness and their chemistry, indicated that their value for materials is as follows: PA, PET, and PP.

ZnO deposition on textile surface by CBD method was preceded by polymer materials covering with nucleus centers in the form of ZnO NPs. The weight gain, XRD and SEM for obtained samples indicate that zinc oxide microrods with the wurzite structure were formed in flower-like agglomerates. A significant influence of a balance between roughness and wetting of textile’s surfaces were demonstrated. The biggest amount of ZnO microrods was present on PA, then PET and the least on PP. Crystallite sizes and strain values are increasing for samples in the following order: PET, PA and PP. In comparison with previous works by the authors, ZnO rods are much smaller, which could be a consequence of using transitional stage of CBD deposition (covering by ZnO NPs). 

It was shown that ZnO modified textiles have a significant antibacterial activity, particularly against Gram-negative bacteria. The highest bactericidal effect was observed for PA-ZnO and this is a consequence of ZnO content, A_R_ values and crystalline parameters that are directly related to ROS generation.

## Figures and Tables

**Table 1:** Characteristic features of used textile superficially modified.

Textile	Warp		Weft		Fabric Surface Mass (g/m^2^)	The Way of Fabric Preparation
	Yarn Character	Number of Threads/10 cm	Yarn Character	Number of Threads/10 cm		
PP	84 dtexf33	460	84 dtexf33	330	72	washing
PET	84 dtexf48	390	150 dtexf216	320	79	washing, thermal stabilization (20 s, 190 °C)
PA	72 dtexF17	380	160 dtexF144	310	81	washing, thermal stabilization (20 s, 185 °C)

**Table 2:** DSC results for analyzed textiles.

Sample	Δ*H_m_*_100_ (J/g)	Δ*H_m_* (J/g)	*X_c_* (%)
PA	230.1	116.2	50.5
PET	140.1	70.7	50.5
PP	207.1	106.1	51.2

**Table 3:** LAC results for analyzed textiles.

Sample	PA	PET	PP
LAC (%)	185 ±	169 ± 3	160 ± 5

**Table 4:** Changes in ZnO unit cell parameters and crystallites.

Sample	d_(100)_ (Å)	d_(002)_ (Å)	a (Å)	c (Å)	V (Å^3^)	*ε*	D (nm)
PA	2.83	2.62	3.27	5.24	48.52	0.00069	23.3
PET	2.83	2.61	3.27	5.23	48.43	0.00061	22.8
PP	2.83	2.61	3.27	5.23	48.43	0.00099	24.6
Standard	2.81	2.60	3.25	5.21	47.60	-	-
